# Recovery potential of cavitation-induced injured cells of common spore-forming bacteria in skim milk exposed to ultrasonication

**DOI:** 10.3168/jdsc.2021-0109

**Published:** 2021-09-13

**Authors:** T.A. Almalki, S. Anand

**Affiliations:** Midwest Dairy Foods Research Center, Dairy and Food Science Department, South Dakota State University, Brookings 57007

## Abstract

The presence of spore-forming microorganisms and their adhesion to contact surfaces in dairy plants is a major concern because dairy products are prone to cross-contamination. Spore formers and their spores can survive milk processing treatments due to their heat resistance. One source of these spore formers is bacterial biofilms, which grow and accumulate on most surfaces in dairy industrial plants, such as pipes, heat exchangers, pasteurized plates, and storage tanks. Their endospores form biofilms by attaching to these surfaces and germinating when conditions become more favorable. The cross-contamination of dairy products by bacterial biofilms may lead to reduced shelf life and spoilage. To minimize the problem caused by thermoduric bacteria, the dairy industry adopts several approaches. Pasteurization is an irreplaceable technique for milk processing. Unfortunately, some bacteria and endospores are resistant to heat treatment, which can grow and cause spoilage of dairy products. Thus, alternative approaches that could help to reduce microbial counts are needed. In our previous study, we demonstrated the effectiveness of ultrasonication to inactivate spore formers and reduce the overall microbial counts in milk. In the current study, we investigated the recovery of cavitation-induced injured cells during the storage of ultrasonicated skim milk. Three common spore formers—*Geobacillus stearothermophilus* (ATCC 15952), *Bacillus licheniformis* (ATCC 6634), and *Bacillus sporothermodurans* (DSM 10599)—were selected to conduct challenge studies by inoculating skim milk samples and exposing them to ultrasonication (10 min each at 80% amplitude). This treatment was done in an ice bath to control the resultant temperature increase. The ultrasonicated skim milk samples were then held for 1, 2, 4, or 12 h in the refrigerator (4°C) to study the recovery of cells following cavitation-induced injury. Ultrasonication resulted in cell injury, as demonstrated by scanning electron microscopy. The injured cells can potentially recover under appropriate conditions during the storage of ultrasonicated milk and could affect the microbiological quality of milk and products manufactured with such milk. The respective bacterial counts for the 3 organisms in the spiked skim milk, on average, were approximately 6.0 log cfu/mL; immediately after ultrasonication, these counts decreased to 3.50 ± 0.02, 4.38 ± 0.02, and 3.75 ± 0.05 log cfu/mL for *G. stearothermophilus*, *B. licheniformis*, and *B. sporothermodurans*, respectively. During 12 h of subsequent incubation at 4°C, their counts increased to 4.17 ± 0.05, 5.25 ± 0.1, and 5.69 ± 0.06 log cfu/mL, respectively. All experiments were done in triplicate for all 3 bacteria. To conclude, slow recovery of injured cells of spore-forming bacteria is possible in ultrasonicated milk during storage under refrigeration conditions.

## Highlights

• Ultrasonication is a new technique that could lower bacterial counts in milk.
• Ultrasonication treatment may cause injury to the bacterial cells.
• Holding the cavitated milk helps the bacteria to recover and return to normalcy.

## Graphical Abstract

**Figure:**
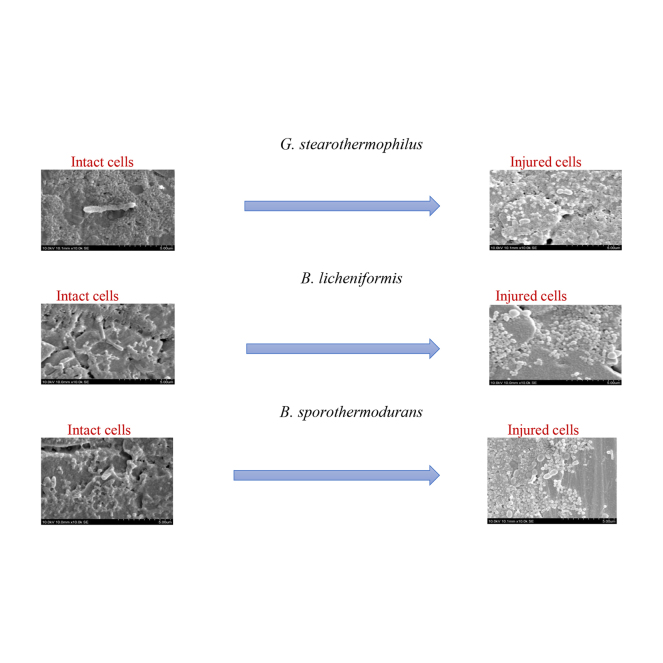
**Summary:** The dairy industry is looking for new techniques to reduce spore-forming organisms that may be present in milk and cause spoilage in milk and its products. Biofilm is the most common source of cross-contamination in dairy manufacturing plants. Ultrasonication is a nonthermal technique that can inactivate spore formers and lower counts of organisms that form biofilm. Ultrasonication induces sound waves above the frequency of human hearing. The sound waves generate thousands of bubbles that quickly collapse, leading to disintegration of the bubbles into small fractions and cause an increase in temperature. Ultrasonication may inactivate or injure spore formers during this process but injured cells can repair themselves within a short time. Our results support the hypothesis that cavitated spore formers can recover and form biofilms.

Thermoduric spore-forming bacteria (spore formers) are responsible for spoilage of several dairy product types. Cross-contamination of dairy products from the processing environment is one of the primary issues that occur in the dairy industry (Andersson et al., 1995). Many studies have been conducted to develop techniques to control, reduce, or eliminate microbial counts and pathogens; these techniques and agents include heating, freezing, ultrasonication, ultra-high pressure, antimicrobial agents, sanitizers, and disinfectants. Pasteurization has been used for decades to kill pathogenic bacteria (Farkas, 1998). We have conducted studies to combine thermal and nonthermal techniques to inactivate or kill common spore formers by applying ultrasonication and pasteurization (Khanal et al., 2014). Although these treatments are quite effective at inactivating bacteria, unfortunately, some of the cells are injured but able to recover.. Exposing common spore formers to several nonthermal treatments such as ultrasonication affects the viability of sporeformers (Wu et al., 2000) and leads to injury and later recovery in dairy products (Deeth and Datta, 2011). The presence of injured cells is as significant as that of the original cells because they can resuscitate and grow in a medium that provides the necessary nutrition under a suitable temperature. Detection of injured cells is thus important in determining food spoilage and safety during the manufacturing of food products (Lado and Yousef, 2002). The importance of detecting injured and uninjured cells cannot be overlooked. However, it is important to differentiate between living cells and dead cells to avoid false-negative and false-positive results.

Injured cells are defined as cells that endure treatment and survive, albeit with loss of certain attributes and changes in component structure and function (Busta, 1976). Providing a specific medium with particular nutrients helps injured cells recover, germinate, and form colonies (Palumbo, 1989). In contrast, dead cells cannot recover from injuries and thus will not form colonies under any condition. Some injured cells can escape food-processing treatments such as heat treatment, freezing, refrigeration, acidity, and water activity (Podolak et al., 2010). In general, most injured vegetative cells recover within 2 to 4 h when incubated at an optimum temperature in a nonselective medium (Wu, 2008). When spore formers are damaged or injured, they lose their normal metabolic activity but regain it after recovery. Several methods exist to recover microorganisms in either a liquid or solid medium (Johnson, 1995). In this study, we used skim milk as a suspension medium to recover injured cells of common spore formers after ultrasonication treatment by holding at 4°C. These injured cells are important because some dairy plants are likely to hold milk for different periods before further processing and, under such situations, injured cells might recover, grow, and lead to spoilage.

Three aerobic spore-forming species were used in this study: *Geobacillus stearothermophilus* (ATCC 15952), *Bacillus licheniformis* (ATCC 6634), and *Bacillus sporothermodurans* (DSM 10599). Pure cultures of these strains were purchased from the American Type Culture Collection (ATCC) and the Deutsche Sammlung von Microorganism and Zellkulturen (German Collection of Microorganisms and Cell Cultures; DSMZ) in Germany. Pure cultures of these strains were grown by incubation at their optimum temperature (55°C, 30°C, and 30°C, respectively) in brain heart infusion (**BHI**) broth (Oxoid/Thermo Scientific).

We selected these species to assess the ability of injured cells to recover after ultrasonication treatment. All 3 spore-forming species were grown in BHI at their optimum temperatures. At the mid-exponential phase, cultures were pelleted by centrifugation at 4,500 × *g* for 30 min, and the pellets were suspended in PBS at pH 7.4. Optical density was adjusted for each organism to reach 0.3, reflecting a concentration of 6 to 7 log cfu/mL. After this step, the organisms were ready to be inoculated in skim milk for further experiments.

One liter of reconstituted nonfat dry milk was prepared for each experiment. Skim milk powder (Associated Milk Producers Inc.) was obtained from the South Dakota State University dairy plant. This reconstituted skim milk was autoclaved at 121°C for 15 min and cooled before being used for the experiments. Sterile skim milk was inoculated at 6 to 7 log cfu/mL of each cell suspension prepared as above.

Ultrasonication was conducted using a 500-W ultrasonic processor (505 Vibra-Cell high intensity; Sonics and Materials Inc.) with a 13-mm stainless steel probe; this probe tends to resonate at 20 kHz. The probe was sanitized with 70% alcohol followed by washing with distilled water before and after conducting each trial. Three to four centimeters of the probe height was inserted vertically in the sample (Cameron et al., 2009). The 3 organisms, *G. stearothermophilus*, *B. licheniformis*, and *B. sporothermodurans*, were separately exposed to ultrasonication (10 min each at 80% amplitude) in 20 mL of skim milk. All trials were repeated in triplicate. Inoculated milk samples were ultrasonicated while submerged in an ice bath to control the temperature increase. A standard enumeration technique was used to determine counts of injured cells.

To study the recovery of the injured cells, ultrasonicated samples were held for 1, 2, 4, or 12 h at refrigeration temperature (4°C). At the end of each holding time, the samples were serially diluted in PBS, and 100 μL of each dilution was spread on prepoured agar plates using sterile plastic spreaders (Fisher Scientific. After spreading, the plates were incubated for 24 h at the respective temperatures, and the colonies were enumerated (Downes et al., 2001). This experiment was conducted as a proof-of-concept study to demonstrate the recovery of the injured cells at the respective temperature; however, further studies need to be done using industrial holding temperatures.

Milk samples of pre- and post-ultrasonication treatment were air-dried on glass slides and observed under scanning electron microscopy, as described by Bulla et al. (1969). The samples were observed using a Hitachi scanning electron microscope model S-3400N; Hitachi SCI Systems Ltd.) at a 30 kV accelerating voltage to observe biofilm from a distance of 8.0 to 8.5 m from the samples. Samples observed before ultrasonication were used as controls to compare the morphology of the vegetative cells before and after ultrasonication treatment.

Every trial was done in triplicate with 3 observations each. The data obtained were analyzed using SAS software (SAS Institute Inc.). Mean values (n = 9) were compared by using one-way ANOVA with a significant difference at *P* < 0.05.

Several micrographs were taken under the scanning electron microscope, before and after ultrasonication at 80% for 10 min, to compare the physical changes in cell morphology. A comparison of cell morphology of the thermoduric species is shown in Figure 1. As can be seen, the morphology of the vegetative cells before ultrasonication was long, rod-shaped, and round-ended cells. After treatment, distortion of the cells could be observed. Vegetative cells disintegrated into several pieces and were shorter with irregular dimensions. Some unaffected cells were apparent under the scanning electron microscope. This experiment demonstrated that ultrasonication could inactivate or injure most vegetative cells in all 3 spore-forming species, in agreement with previous studies. Prior investigations indicated similar structural changes to cells after ultrasonication in such as *Escherichia coli* and *Streptococcus mutans* (Lee et al., 2009).

**Figure 1:**
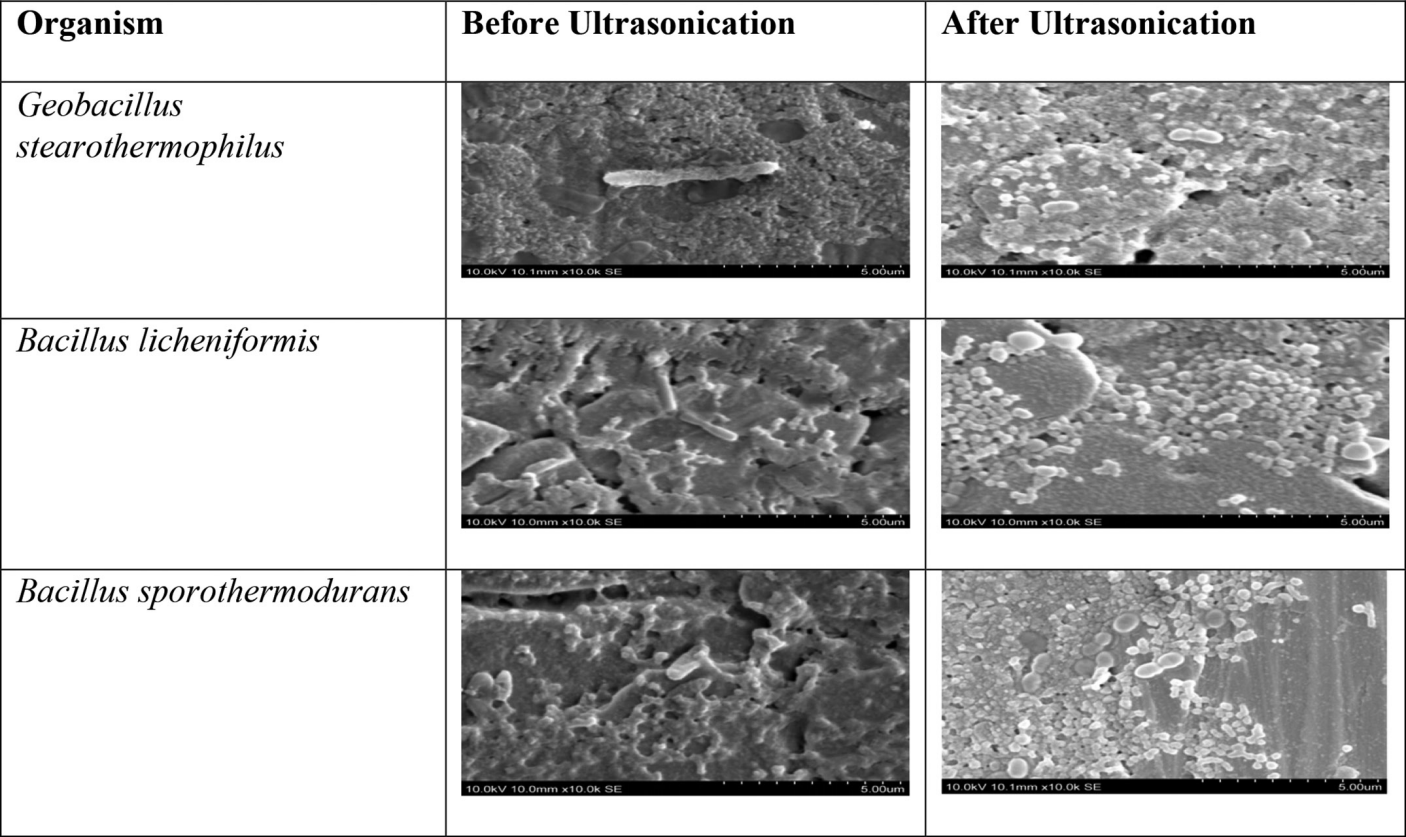
Scanning electron micrographs showing vegetative cell morphology of 3 spore-forming organisms before and after ultrasonication. Note the changes in the shape of the cells, which appear as long rods before treatment and as shorter beaded structures after treatment.

In this experiment, the skim milk samples were spiked with bacterial vegetative cells at 6 to 7 logs. Immediately after ultrasonication at 80% for 10 min in an ice bath, bacterial counts were assessed (0 h). After plating and incubating for 24 to 48 h, the 0-h counts were recorded (Table 1). *Geobacillus stearothermophilus* showed an average count of 3.50 ± 0.025 log cfu/mL. However, *B. licheniformis* and *B. sporothermodurans* showed counts of 4.38 ± 0.021 and 3.75 ± 0.055 log cfu/mL under the same processing parameters. The extent of reduction in spore former counts as a result of ultrasonication was comparable to that of our previous study (Khanal et al., 2014).

**Table 1:** Mean counts (log cfu/mL) of sporeformers during incubation at 4°C after ultrasonication treatment

Organism	Incubation time				
	0 h	1 h	2 h	4 h	12 h
*Geobacillus stearothermophilus*	3.5 ± 0.014^a^	3.64 ± 0.02^b^	3.79 ± 0.01^c^	3.95 ± 0.01^d^	4.17 ± 0.05^e^
*Bacillus licheniformis*	4.38 ± 0.02^a^	4.82 ± 0.02^b^	5.05 ± 0.03^c^	5.19 ± 0.02^d^	5.52 ± 0.1^e^
*Bacillus sporothermodurans*	3.75 ± 0.003^a^	4.37 ± 0.01^b^	4.95 ± 0.01^c^	5.24 ± 0.01^d^	5.69 ± 0.06^e^

a–e Values with different superscript letters within a row are significantly different at *P* < 0.05.

The results presented in Table 1 show the effect of holding in the refrigerator after ultrasonication on the repair and recovery of the 3 thermoduric species for different durations. The mean 0-h log count for *G. stearothermophilus* was 3.50 ± 0.05 log cfu/mL, which increased to 4.17 ± 0.05 log cfu/mL after 12 h of holding at 4°C. The optimal growth of *G. stearothermophilus*, *B. licheniformis*, and *B. sporothermodurans* was 10^7^, 10^6^, and 10^6^ logs, respectively. The results in Table 1 show a slow recovery of cells and indicate that injured cells may not have the ability to repair quickly in skim milk. This pattern of recovery was comparable for all the 3 trials carried out using *G. stearothermophilus*.

The ability of *B. licheniformis* to recover after ultrasonication treatment is also shown in Table 1. A higher recovery of cells can be seen by the mean 12-h count of 5.52 log cfu/mL, indicating a >1.0 log increase in counts after 12 h at 4°C (*P* < 0.05). The comparable data across triplicate trials help establish the reproducibility of these experiments. The observed increase in counts demonstrated the proof of concept for the greater recovery of injured *B. licheniformis* cells compared with injured cells of *G. stearothermophilus.* Although no other studies are available to compare the recovery of cavitated cells of spore formers under similar conditions in skim milk, studies in other organisms provide evidence of recovery on selective agar.

Although no direct reference is available to compare the recovery potential of injured cells, some previous studies have reported recovery from injury assessed using different methods and recovery media. Injured cells could recover in just 1 h at 25°C in a nonselective medium such as tryptic soy broth (Todd et al., 1988). In another study, acid-injured cells of *E. coli* were recovered and enumerated using hydrophobic grid membrane filtration in combination with agar (Kang and Siragusa, 1999). Kang and Siragusa (1999) also used the agar underlay method for recovery of sublethal heat–injured bacteria such as *E. coli* and *Salmonella typhimurium* and found that recovery of these species was similar on selective and nonselective agar. However, Kang and Fung (2000) conducted a study to evaluate the recovery of injured *S. typhimurium* in 2 different media, xylose lysine decarboxylase (XLD) and tryptic soy agar (TSA), at 55°C for 15 min. After a few hours of incubation, *Salmonella typhimurium* recovered and grew more in XLD than in TSA.

Table 1 shows results for recovery of *B. sporothermodurans* following ultrasonication. The count immediately after treatment was 3.75 logs, which increased to 4.37 logs within 1 h. The greatest count, 5.69 logs, was recorded at 12 h. Thus, injured cells of *B. sporothermodurans* showed the greatest recovery of the 3 species evaluated. Different bacteria exhibit different responses to the treatment they are exposed to and in their ability to repair injured cells. Some will be eliminated, but others may recover as injured cells, leading to full growth under favorable conditions. In a previous study, skim milk, whole milk, and peptone sorbitol bile broth were inoculated with *Yersinia enterocolitica* and pasteurized at 62.8°C for 30 min. After 8 d of incubation at 10°C, bacteria were recovered in peptone sorbitol bile broth. However, recovery in the other media was slower than that in peptone and likely would not present a hazard after storage at low temperature (Kushal and Anand, 1999).

This study presents a proof of concept of the ability of ultrasonicated injured spore-forming bacteria to recover within 4 h in skim milk. Based on this work, further studies should be conducted with multispecies inoculants using industrial milk-holding temperatures for broader application. Our results indicate that skim milk is a suitable medium for the recovery and repair of spore formers injured by nonthermal processes such as ultrasonication. This study provides useful information on the use of cavitation as part of the process to partially inactivate thermoduric spore formers.

## References

[bib1] Andersson A., Rönner U., Granum P.E. (1995). What problems does the food industry have with the spore-forming pathogens *Bacillus cereus* and *Clostridium perfringens*?. Int. J. Food Microbiol..

[bib2] Bulla L., Julian G.S., Rhodes R., Hesseltine C. (1969). Scanning electron and phase-contrast microscopy of bacterial spores. Appl. Microbiol..

[bib3] Busta F. (1976). Practical implications of injured microorganisms in food. J. Milk Food Technol..

[bib4] Cameron M., McMaster L.D., Britz T.J. (2009). Impact of ultrasound on dairy spoilage microbes and milk components. Dairy Sci. Technol..

[bib5] Deeth H., Datta N., Fuquay J.W., Fox P.F., McSweeney P.L.H. (2011). Encyclopedia of Dairy Sciences.

[bib6] Downes J., Munson M.A., Spratt D.A., Kononen E., Tarkka E., Jousimies-somer H., Wade W.G. (2001). Characterization of *Eubacterium*-like strains isolated from oral infections. J. Med. Microbiol..

[bib7] Farkas J. (1998). Irradiation as a method for decontaminating food: A review. Int. J. Food Microbiol..

[bib8] Johnson D. (1995). Selective solid media for isolating and enumerating acidophilic bacteria. J. Microbiol. Methods.

[bib9] Kang D.-H., Fung D.Y. (2000). Application of thin agar layer method for recovery of injured *Salmonella* typhimurium. Int. J. Food Microbiol..

[bib10] Kang D.H., Siragusa G. (1999). Agar underlay method for recovery of sublethally heat-injured bacteria. Appl. Environ. Microbiol..

[bib11] Khanal S.N., Anand S., Muthukumarappan K., Huegli M. (2014). Inactivation of thermoduric aerobic spore formers in milk by ultrasonication. Food Control.

[bib12] Kushal R., Anand S.K. (1999). Repair and recovery of thermally injured cells of *Yersinia enterocolitica* in milk. J. Food Prot..

[bib13] Lado B.H., Yousef A.E. (2002). Alternative food-preservation technologies: efficacy and mechanisms. Microbes Infect..

[bib14] Lee H., Zhou B., Liang W., Feng H., Martin S.E. (2009). Inactivation of *Escherichia coli* cells with sonication, manosonication, thermosonication, and manothermosonication: Microbial responses and kinetics modeling. J. Food Eng..

[bib15] Palumbo S.A., Ray B. (1989). Injured Index and Pathogenic Bacteria: Occurrence and Detection in Foods, Water and Feeds.

[bib17] Podolak R., Enache E., Stone W., Black D.G., Elliott P.H. (2010). Sources and risk factors for contamination, survival, persistence, and heat resistance of *Salmonella* in low-moisture foods. J. Food Prot..

[bib18] Todd E.C., Szabo R., Peterkin P., Sharpe A., Parrington L., Bundle D., Gidney M., Perry M. (1988). Rapid hydrophobic grid membrane filter-enzyme-labeled antibody procedure for identification and enumeration of *Escherichia coli* O157 in foods. Appl. Environ. Microbiol..

[bib19] Wu H., Hulbert G.J., Mount J.R. (2000). Effects of ultrasound on milk homogenization and fermentation with yogurt starter. Innov. Food Sci. Emerg. Technol..

[bib20] Wu V.C. (2008). A review of microbial injury and recovery methods in food. Food Microbiol..

